# The evaluation of pretreatment neutrophil–lymphocyte ratio and derived neutrophil–lymphocyte ratio in patients with laryngeal neoplasms^☆^

**DOI:** 10.1016/j.bjorl.2018.04.013

**Published:** 2018-06-08

**Authors:** Gorkem Eskiizmir, Uzdan Uz, Ece Onur, Beyhan Ozyurt, Gizem Karaca Cikrikci, Nevin Sahin, Arzu Oran, Onur Celik

**Affiliations:** a Manisa Celal Bayar University, Department of Otolaryngology-Head and Neck Surgery, Manisa, Turkey; b University of Health Sciences, Izmir Bozyaka Training and Research Hospital, Department of Otolaryngology-Head and Neck Surgery, Izmir, Turkey; c Manisa Celal Bayar University, Department of Biochemistry, Manisa, Turkey; d Manisa Celal Bayar University, Department of Public Health, Manisa, Turkey

**Keywords:** Laryngeal neoplasm, Neutrophil, Lymphocyte, Biomarker, Prognosis, Survival, Neoplasia laríngea, Neutrófilo, Linfócito, Biomarcador, Prognóstico, Sobrevivência

## Abstract

**Introduction:**

Systemic inflammatory biomarkers are promising predictive and prognostic factors for solid cancers. The neutrophil–lymphocyte ratio and derived neutrophil–lymphocyte ratio are used to predict inflammation and used as biomarker in several malignancies.

**Objective:**

The purpose of this study was to demonstrate the diagnostic, predictive and prognostic role of neutrophil–lymphocyte ratio and derived neutrophil–lymphocyte ratio in patients with laryngeal neoplasms.

**Methods:**

A retrospective study was conducted on medical records involving 229 patients with benign, premalignant and malignant laryngeal neoplasms between 2002 and 2015. The diagnostic, predictive and prognostic role of neutrophil–lymphocyte ratio and derived neutrophil–lymphocyte ratio were evaluated using uni– and multivariate analysis.

**Results:**

The neutrophil–lymphocyte ratio and derived neutrophil–lymphocyte ratio were not statistically different between patients with benign, premalignant and malignant laryngeal neoplasms. Both neutrophil–lymphocyte ratio and derived neutrophil–lymphocyte ratio were predictive factors for stage, lymph node metastasis, and distant metastasis. Patients with high neutrophil–lymphocyte ratio value (≥4) had a poor prognosis when compared with patients with low neutrophil–lymphocyte ratio value (5 year, Overall Survival: 69.0% vs. 31.1%, *p* < 0.001; 5 year, disease free survival: 70.0% vs. 32.7%, *p* ˂ 0.001; 5 year, locoregional recurrence free survival: 69.7% vs. 32.0%, *p* < 0.001). Furthermore, neutrophil–lymphocyte ratio was an independent prognostic factor for 5 year: Overall survival (HR = 2.396; 95% CI 1.408–4.077; *p* = 0.001), Disease free survival (HR = 2.246; 95% CI 1.322–3.816; *p* = 0.006) and locoregional recurrence free survival (HR = 2.210; 95% CI 1.301–3.753; *p* = 0.003).

**Conclusion:**

Pretreatment neutrophil–lymphocyte ratio is a useful and reliable predictive and prognostic biomarker for patients with laryngeal carcinoma.

## Resumo

**Introdução:**

Biomarcadores inflamatórios sistêmicos são fatores preditivos e prognósticos promissores para cânceres sólidos. A relação neutrófilo-linfócito e a relação neutrófilo-linfócito derivada são utilizadas para predizer a inflamação e como biomarcadores em várias malignidades.

**Objetivo:**

O objetivo deste estudo foi demonstrar o papel diagnóstico, preditivo e prognóstico da relação neutrófilo-linfócito e relação neutrófilo-linfócito derivada em pacientes com neoplasias laríngeas.

**Método:**

Foi realizado um estudo retrospectivo em prontuários médicos de 229 pacientes com neoplasias laríngeas benignas, pré-malignas e malignas entre 2002 e 2015. O papel diagnóstico, preditivo e prognóstico da relação neutrófilo-linfócito e relação neutrófilo-linfócito derivada foi avaliado por meio de análise uni- e multivariada.

**Resultados:**

A relação neutrófilo-linfócito e a relação neutrófilo-linfócito derivada não foram estatisticamente diferentes entre pacientes com neoplasias laríngeas benignas, pré-malignas e malignas. Ambas as relação neutrófilo-linfócito e relação neutrófilo-linfócito derivada foram fatores preditivos para o estágio, metástase linfonodal e metástase a distância. Pacientes com valor alto da relação neutrófilo-linfócito (≥4) apresentaram pior prognóstico quando comparados com pacientes com valor mais baixo da relação neutrófilo-linfócito (5 anos, Sobrevida Global: 69,0% vs. 31,1%, *p* < 0,001; 5 anos, sobrevida livre de doença: 70,0% vs. 32,7%, *p* < 0,001; 5 anos, sobrevida livre de recorrência loco-regional: 69,7% vs. 32,0%, *p* < 0,001). Além disso, a relação neutrófilo-linfócito foi um fator prognóstico independente para 5 anos: Sobrevida global (HR = 2,396; IC95% 1,408–4,077; p = 0,001), sobrevida livre de doença (HR = 2,246; IC95%: 1,322–3,816; *p* = 0,006) e sobrevida livre de recorrência loco-regional (HR = 2,210; IC95%: 1,301–3,753; *p* = 0,003).

**Conclusão:**

A relação neutrófilo-linfócito no pré-tratamento é um biomarcador preditivo e de prognóstico útil e confiável para pacientes com carcinoma de laringe.

## Introduction

Larynx cancer is one of the most common cancers of head and neck. In 2012, the estimated new cases with laryngeal carcinoma (LC) was 157,000 and cancer related-deaths was 83,000 worldwide.^1^ The five-year survival rate is approximately 75% in patients with localized disease; however, a remarkable decrease in survival is observed when there is regional and distant disease (44% and 35%).^2^ Therefore, early detection of LC, and assessment of regional and distant metastasis (DM) are crucial for clinical staging, treatment selection, locoregional control, and survival.

In the literature, various factors have been reported as diagnostic, predictive and/or prognostic biomarkers for cancer. Cancer biomarkers are unique molecular signatures that are produced by the tumor itself or in response to tumor activity. An ideal cancer biomarker should have following features: (i) highly specific and sensitive, (ii) simple for identification of the disease, (iii) easy to obtain, (iv) stable with gender, age, and ethnicity, (v) indicator of prognosis, and (vi) cost–effective. Despite several biomarkers such as ERRC1, p16, K-ras, EGFR and EGFRvIII, none of the aforementioned biomarkers is routinely and widely used in head and neck cancers because of their limitations in clinical applications, reproducibility, and expense.^3^, ^4^

The neutrophil–lymphocyte ratio (NLR) and derived NLR (dNLR) are recently popularized as systemic inflammatory response biomarkers.^5^, ^6^ Neutrophil–lymphocyte ratio principally determines the systemic inflammation, particularly in chronic inflammatory diseases.^7^, ^8^ Several clinical studies have demonstrated that a high NLR value is associated with poor prognosis and survival in several cancers such as nasopharyngeal, gastrointestinal, lung and renal cancers.^9^, ^10^, ^11^, ^12^ In addition, dNLR, a modified form of NLR, is also used to demonstrate the systemic inflammation and prognosis in a variety of cancers such as gastrointestinal and breast cancers.^6^, ^13^, ^14^ The pathophysiological relationship between high NLR or dNLR values and poor prognosis still remains unclear. It is hypothesized that it may be related to a decrease in the number of lymphocytes and increase in neutrophil counts in patients with cancer.^15^ In fact, lymphocytes are responsible for anticancer immunity response, and CD8(+) T cells specifically control the tumor activity by apoptosis and cytotoxic effect. Therefore, lymphocyte counts are inversely correlated with severity of cancer.^16^, ^17^ Cancer-related inflammation may also lead to an increase in number of neutrophils.^18^, ^19^ Furthermore, cytokines, which are produced by cancerous cells, may trigger the migration of neutrophils from blood to tumor microenvironment; thus, neutrophils may stimulate the tumor growth and angiogenesis by vascular endothelial growth factor, IL-8 and matrix metalloproteinase-9.^20^, ^21^, ^22^

In the literature, the predictive and prognostic role of NLR have been reported in head and neck cancers.^23^, ^24^, ^25^, ^26^ Rassouli et al.^23^ emphasized the relationship between NLR values (>4.27) and risk of tumor recurrence. Moreover, NLR was reported as an independent predictive and prognostic factor for survival in patients with recurrent or metastatic head and neck cancers.^24^ Similarly, Haddad et al.^25^ determined a high risk of mortality in patients with a NLR of ≥5 (89% vs. 61%, *p* = 0.0017). Rachidi et al.^26^ also demonstrated a significant relationship between increased NLR value and poor prognosis in patients with head and neck cancers. They advocated that high level of neutrophils and/or low level of lymphocytes were correlated with a remarkable decrease in overall survival (OS). The purpose of this study was to determine the diagnostic role of pretreatment NLR and dNLR in patients with laryngeal neoplasms, and demonstrate their predictive and prognostic role in patients with LC.

## Methods

This study was approved by the Institutional Ethics Committee of Manisa Celal Bayar University (Approval Number: 20478486-408).

### Patients and methods

A retrospective review was conducted between 2002 and 2015 at Manisa Celal Bayar University, and patients with a diagnosis of laryngeal benign, premalignant and malignant neoplasms were enrolled. The exclusion criteria were as follows: (i) patients with second primary cancer, (ii) patients with chronic inflammatory/autoimmune disorder, (iii) patients with malignant neoplasms other than squamous cell carcinoma. Hence, the study population included three groups: (i) patients with benign laryngeal neoplasms, (ii) patients with premalignant neoplasms, and (iii) patients with malignant neoplasms or LC. All cases were evaluated by clinical and radiological examinations such as ultrasonography, computed tomography or magnetic resonance imaging. TNM stages of patients with LC were determined according to the criteria of the 7th edition of “American Joint Committee on Cancer” Cancer Staging Manual established in 2009.

All samples for complete blood count were collected 5–10 days before the surgical biopsy/excision, which was performed under general anesthesia. Therefore, none of the patients had any sign of infection. The NLR value was measured by dividing the complete neutrophil count (CNC) to complete lymphocyte count (CLC), and the value of dNLR was determined by the formula of CNC/Complete white blood cell count (CWBCC)-CNC.

### Statistical analysis

All statistical analysis was performed using SPSS v. 20.0 for Windows (Chicago, IL, USA). The mean values and standard deviations were calculated according to sociodemographic and clinicopathological characteristics of study population. Kolmogorov Smirnov and Shapiro–Wilk's tests demonstrated an abnormal distribution of NLR and dNLR values. Therefore, Mann–Whitney *U* test was performed for the comparison of two groups; and Kruskal–Wallis test was used for the comparison of three or more groups. In addition, the study population was dichotomized according to cut-off values of NLR and dNLR (low vs. high). The cut-off values were 4 for NLR and 2 for dNLR depending on clinical studies with high evidence level.^5^, ^6^ Thereby, the diagnostic and predictive role of NLR and dNLR was also analyzed using Chi-Square test.

In this study, three endpoints (5 year: OS, disease free survival [DFS] and locoregional recurrence free survival [LRFS]) were selected for the assessment of oncological outcomes. All time to events were measured from the date of initial treatment modality. Overall survival was defined as the time from diagnosis to last follow-up or death for any cause. Disease-free survival was defined as the time from diagnosis to any sign of disease, locoregional recurrence (LR) and/or DM, last follow-up or death for any cause. Locoregional recurrence free survival was defined as the time from treatment to any sign of LR or last followup. Survival analysis was performed by Kaplan–Meier method and variables were compared using log-rank test. A Cox proportional hazard regression model was created in order to determine the effect of variables on oncological outcomes for both uni- and multivariate analysis. The variables with a probability value of <0.1 in the univariate analysis were selected for multivariate proportional Cox hazards regression analysis. Thereafter, only the variables with significance retained in the model using stepwise backward elimination; thereby independent prognostic factors were determined. The results were expressed as hazard ratios (HRs) with 95% confidence interval (CI). A value of *p* < 0.05 was considered statistically significant.

## Results

### Sociodemographic and clinicopathologic characteristics of study population

The study population included 229 patients with laryngeal neoplasms. The descriptive statistics about patient, clinical, histopathological and treatment characteristics were presented in Table 1. The number of patients with benign, premalignant and malignant neoplasms was 30 (17 vocal fold polyps and 13 vocal fold nodules) (13.1%), 13 (9 mild dysplasia and 4 moderate dysplasia) (5.7%), 186 (81.2%), respectively. The majority of cases were male (221 cases, 96.5%). The median age of patients at the time of treatment was 59.0 (range 31–88) years (<65 years, 152 cases, 66.4%). A vast majority of laryngeal malignant neoplasms were glottic (82 cases; 44.1%) and supraglottic (79 cases, 42.5%). Transglottic larynx cancer was found in 24 cases (13.4%). Well, moderately, poorly differentiated and unidentified LC were seen in 25 (13.4%), 95 (51.1%), 15 (8.1%) and 51 (27.4) patients, respectively. Ninety-eight patients (52.7%) were advanced-stage LC, Stage III: 40 cases (21.5%); Stage IV: 58 cases (31.2%) and 88 patients (47.3%) were early-stage LC, Stage I: 48 cases (25.8%); Stage II: 40 cases (21.5%). thyroid cartilage invasion (TCI) was detected in 40 cases (21.5%). The distribution of T-stage I, II, III and IV were 26.3% (49 cases), 22% (41 cases), 28% (52 cases), 23.7% (44 cases), respectively. Lymph node metastasis (LNM) was found in 42 cases (22.6%), N1: 12 cases (28.5%); N2: 30 cases (71.5%) and extracapsular nodal spread was detected in 26 patients (61.9%). Seven patients (3.8%) with LC had DM at the time of diagnosis. Patients with early-stage LC were mainly treated with radiotherapy (57 cases, 64.8%). On the other hand, most of the patients with advanced-stage LC had surgical treatment and adjuvant radio ± chemotherapy (67 cases, 68.4%). Ninety-eight patients (52.7%) were treated with surgery ± adjuvant therapy and 88 cases (47.3%) with non-surgical modalities. The mean value of follow-up was 39.5 (1–107) months. During follow-up, LR and DM were detected in 33 cases (18.4%) and 19 cases (10.6%), respectively. Sixty-five patients (34.9%) died during the follow-up period.

**Table 1:** Patient and disease characteristics, predictive analysis of NLR and dNLR with demographic and clinicopathological characteristics.

	Patients grouped by NLR level (*n* %)		*p*	Patients grouped by dNLR level (*n* %)		*p*
	Low (< 4)	High (≥ 4)		Low (< 2)	High (≥ 2)	
*Age*			0.685			0.909
<65 years (*n* = 152)	123 (80.9)	29 (19.1)		92 (60.5)	60 (39.5)	
>65 years (*n* = 77)	64 (83.1)	13 (16.9)		46 (59.7)	31 (40.3)	

*Gender*			0.641			1.000
Female (*n* = 8)	6 (75.0)	2 (25.0)		5 (62.5)	3 (37.5)	
Male (*n* = 221)	181 (81.9)	40 (18.1)		133 (60.2)	88 (39.8)	

*Study group*			0.232			0.399
Benign (*n* = 30)	27 (90.0)	3 (10.0)		133 (60.2)	88 (39.8)	
Premalignant (*n* = 13)	12 (92.3)	1 (7.7)		133 (60.2)	88 (39.8)	
Malignant (*n* = 186)	148 (79.6)	38 (20.4)		133 (60.2)	88 (39.8)	

*Location of larynx cancer*			<0.001			0.001
Supraglottic (*n* = 79)	55 (69.6)	24 (30.4)		40 (50.6)	39 (49.4)	
Glottic (*n* = 82)	77 (93.9)	5 (6.1)		60 (73.2)	22 (26.8)	
Transglottic (*n* = 25)	16 (64)	9 (36.0)		9 (36.0)	16 (64.0)	

*TNM stage*			<0.001			0.008
Stage I (*n* = 48)	46 (95.8)	2 (4.2)		36 (75.0)	12 (25.0)	
Stage II (*n* = 40)	34 (85.0)	6 (15.0)		26 (65.0)	14 (35.0)	
Stage III (*n* = 40)	33 (82.5)	7 (17.5)		22 (55.0)	18 (45.0)	
Stage IV (*n* = 58)	35 (60.3)	23 (39.7)		25 (43.1)	33 (56.9)	

*Stage*			<0.001			0.002
Early-stage (*n* = 88)	80 (90.9)	8 (9.1)		62 (70.5)	26 (29.5)	
Advanced-stage (*n* = 98)	68 (69.4)	30 (30.6)		47 (48.0)	51 (52.0)	

*T-stage*			<0.001			0.004
T1 (*n* = 49)	47 (95.9)	2 (4.1)		37 (75.5)	12 (24.5)	
T2 (*n* = 41)	35 (85.4)	6 (14.6)		27 (65.9)	14 (34.1)	
T3 (*n* = 52)	39 (75.0)	13 (25.0)		27 (51.9)	25 (48.1)	
T4 (*n* = 44)	27 (61.4)	17 (38.6)		18 (40.9)	26 (59.1)	

*Thyroid cartilage invasion*			<0.001			0.002
Absent (*n* = 146)	125 (85.6)	21 (14.4)		93 (63.7)	53 (36.3)	
Present (*n* = 40)	23 (57.5)	17 (42.5)		16 (40.0)	24 (60.0)	

*Grade*			0.890			0.073
Well-differentiated (*n* = 25)	21 (84)	4 (16)		19 (76.0)	6 (24.0)	
Moderately differentiated (*n* = 95)	69 (72.6)	26 (27.4)		48 (50.5)	47 (49.5)	
Poor differentiated (*n* = 15)	13 (86.7)	2 (13.3)		8 (53.3)	7 (46.7)	

*N-stage*			<0.001			0.075
N0 (*n* = 144)	123 (85.4)	21 (14.6)		90 (62.5)	54 (37.5)	
N1 (*n* = 12)	9 (75.0)	3 (25.0)		7 (58.3)	5 (41.7)	
N2 (*n* = 40)	16 (53.3)	14 (46.7)		12 (40.0)	18 (60.0)	

*Lymph node metastasis*			<0.001			0.046
Absent (*n* = 144)	123 (85.4)	21 (14.6)		90 (62.5)	54 (37.5)	
Present (*n* = 52)	25 (59.5)	17 (40.5)		19 (45.2)	23 (54.8)	

*M-stage*			0.004			0.021
M0 (*n* = 179)	146 (81.6)	33 (18.4)		108 (60.3)	71 (39.7)	
M1 (*n* = 7)	2 (28.6)	5 (71.4)		1 (14.3)	6 (85.7)	

### The correlation of NLR and dNLR with sociodemographic and clinicopathologic characteristics

**Figure 1:**
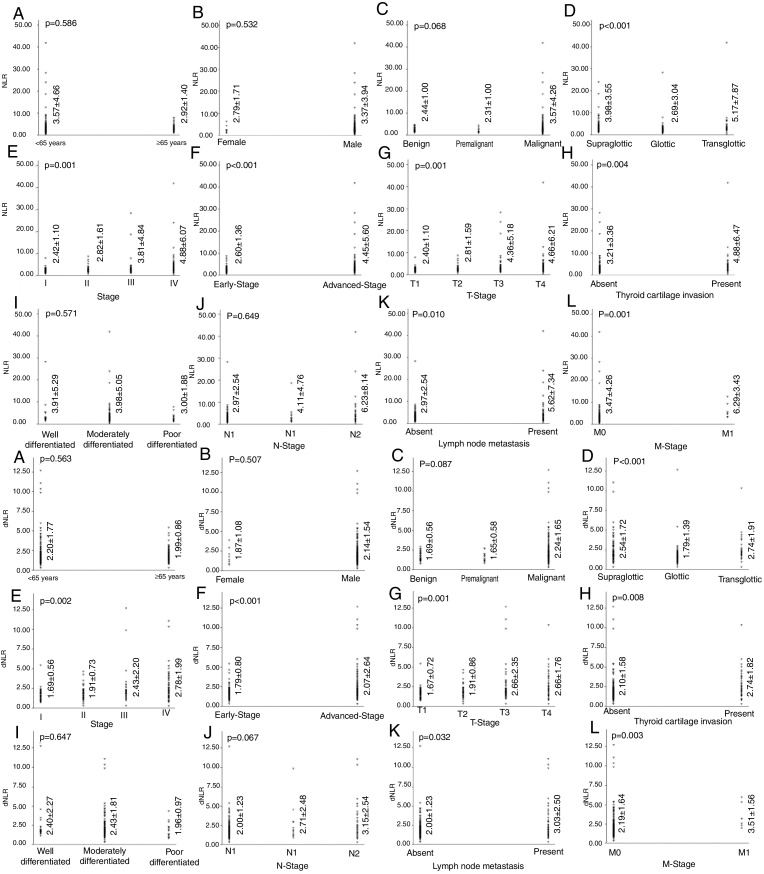
The distribution, mean values, standard deviations and p-values of NLR and dNLR according to sociodemographic and clinicopathologic characteristics.

The distribution, mean values and standard deviations of NLR and dNLR are presented in Fig. 1. The mean values of NLR and dNLR were relatively high in patients with LC; however, the difference was not statistically significant when patients with laryngeal benign, premalignant and malignant neoplasms were compared (*p* = 0.068 and *p* = 0.087) (Fig. 1). Similarly, no statistically significant difference was determined between patients with LC and other groups when the study groups compared according to low versus high NLR and dNLR values (*p* = 0.232 and *p* = 0.399) (Table 1). On the other hand, high NLR and dNLR values were highly correlated with supraglottic/transglottic, advanced stage LC and presence of TCI, LNM and DM (Table 1) (Fig. 1).

### The association between NLR, dNLR and oncological outcomes: Uni- and multivariate analysis

**Figure 2:**
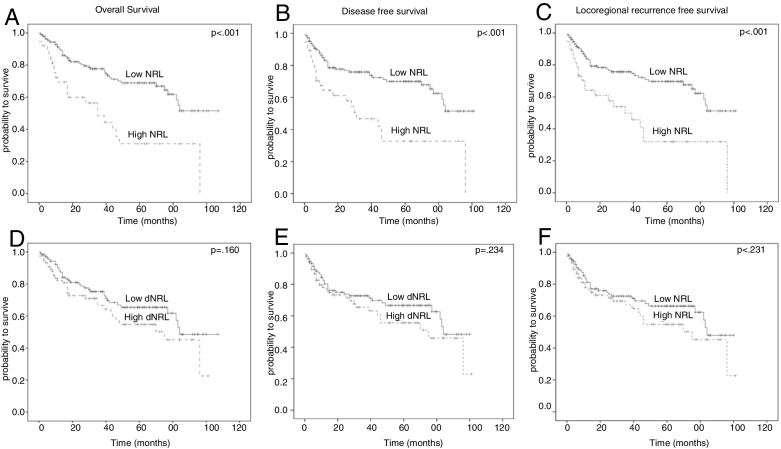
Log rank analysis of NLR and dNLR for 5 year OS, DFS and LRFS.

Five-year OS, DFS and LRFS of patients with LC were 61.3%, 62.3% and 61.8%, respectively. Patients with NLR ≥ 4 had significantly poor oncological outcomes when compared with NLR < 4; 5 year (OS: 69.0% vs. 31.1%, *p* < 0.001; DFS: 70.0% vs. 32.7%, *p* < 0.001; LRFS: 69.7% vs. 32.0%, *p* < 0.001) (Fig. 2A–C). However, no statistically significant difference between low and high dNLR values was determined for 5 year: OS (65.4% vs. 54.8%, *p* = 0.160); DFS (66.6% vs. 55.5%, *p* = 0.234) and LRFS (66.3% vs. 54.8%, *p* = 0.231) (Fig. 2D–F). Moreover, multivariate analysis using stepwise backward elimination demonstrated that NLR was an independent prognostic factor for 5 year: OS (HR = 2.396, 95% CI 1.408–4.077; *p* = 0.001); DFS (HR = 2.246, 95% CI 1.322–3.816; *p* = 0.006) and LRFS (HR = 2.210, 95% CI 1.301–3.753; *p* = 0.003) (Table 2).

**Table 2:** Uni- and multivariate analysis for 5-year overall survival, disease specific survival, disease free survival, locoregional recurrence free survival and distant metastasis free survival.

	Overall survival				Disease free survival				Locoregional recurrence free survival			
	Univariate analysis		Multivariate analysis		Univariate analysis		Multivariate analysis		Univariate analysis		Multivariate analysis	
	HR (95% CI)	*p*	HR (95% CI)	*p*	HR (95% CI)	*p*	HR (95% CI)	*p*	HR (95% CI)	*p*	HR (95% CI)	*p*
*Age*		0.011		0.005		0.011		0.003		0.010		0.003
<65 years	1 (Reference)		1 (Reference)		1 (Reference)		1 (Reference)		1 (Reference)		1 (Ref.)	
≥65 years	1.889 (1.154–3.092)		2.045 (1.246–3.357)		1.891 (1.155–3.095)		2.001 (1.220–3.282)		1.918 (1.172–3.139)		2.210 (1.301–3.753)	

*Stage*		0.002		0.295		0.005		0.547		0.006		0.584
Early-stage	1 (Reference)		1 (Reference)		1 (Reference)		1 (Reference)		1 (Reference)		1 (Ref.)	
Advanced-stage	2.289 (1.360–3.855)		1.410 (0.741–2.684)		2.112 (1.255–3.556)		1.239 (0.618–2.483)		2.077 (1.234–3.496)		1.215 (0.606–2.437)	

*Thyroid cartilage invasion*		< 0.001		<0.001		< 0.001		<0.001		<0.001		<0.001
Absent	1 (Reference)		1 (Reference)		1 (Reference)		1 (Reference)		1 (Reference)		1 (Reference)	
Present	3.462 (2.092–5.727)		3.159 (1.889–5.281)		3.119 (1.885–5.159)		2.825 (1.691–4.721)		3.129 (1.892–5.175)		2.803 (1.677–4.684)	

*Lymph node metastasis*		0.042		0.549		0.062		0.333		0.072		0.346
Absent	1 (Reference)		1 (Reference)		1 (Reference)		1 (Reference)		1 (Reference)		1 (Reference)	
Present	1.730 (1.020–2.935)		1.220 (0.636–2.339)		1.657 (0.976–2.813)		1.349 (0.736–2.474)		1.626 (0.958–2.760)		1.337 (0.731–2.445)	

*NLR*		< 0.001		0.001		0.001		0.006		0.001		0.003
Low	1 (Reference)		1 (Reference)		1 (Reference)		1 (Reference)		1 (Reference)		1 (Reference)	
High	2.675 (1.597–4.483)		2.396 (1.408–4.077)		2.500 (1.492–4.189)		2.246 (1.322–3.816)		2.498 (1.491–4.185)		2.210 (1.301–3.753)	

*dNLR*		0.163		0.302		0.238		0.272		0.235		0.271
Low	1 (Reference)		1 (Reference)		1 (Reference)		1 (Reference)		1 (Reference)		1 (Reference)	
High	1.415 (0.868–2.307)		0.666 (0.308–1.440)		1.342 (0.823–2.188)		0.649 (0.300–1.403)		1.344 (0.825–2.191)		0.648 (0.300–1.402)	

## Discussion

To the best of our knowledge, this is the first study in which the diagnostic, predictive and prognostic role of both NLR and dNLR were comprehensively analyzed in patients with laryngeal neoplasms. The study population and duration of follow-up are unique to determine the relationship between these systemic inflammatory response biomarkers and laryngeal neoplasms. Our literature survey showed that the relationship between dNLR and laryngeal neoplasms has not been evaluated, and few numbers of clinical studies assessed the correlation between NLR and laryngeal neoplasms (Table 3).^27^, ^28^, ^29^, ^30^, ^31^, ^32^, ^33^, ^34^ In this study, no statistically significant difference was determined when a comparison between patients <65 years and ≥65 years was performed (Table 1) (Fig. 1). In addition, NLR and dNLR values were not statistically different in male and female patients (Table 1) (Fig. 1). Therefore, our results indicated that both NLR and dNLR were not affected by age and gender, which is a prerequisite for a cancer biomarker.

**Table 3:** Review of clinical studies in which predictive and prognostic role of NLR were evaluated in patients with LSCC.

Year	Author	*n*	Study	Survival parameters	NLR grouping	NLR cut-off value	Conclusion
2014	Kum et al. ^27^	209 Benign, Premalignant, Malignant	Comparison of groups	–	–	–	NLR was significantly higher in patients with laryngeal cancer than other groups.
2015	Duzlu et al. ^28^	65 LSCC, 42 Control	Comparison of groups	–	–	–	NLR was significantly higher in patients with laryngeal cancer than control group.
2015	Tu et al. ^29^	141 LSCC	Prognostic evaluation	Disease free survival, overall survival	Receiver Operating Characteristic (ROC) Curve Analysis	2.17	High NLR was a prognostic factor for overall- and disease free survival.
2016	Wong et al. ^30^	140 LSCC	Prognostic evaluation	Disease free survival, overall survival	Quartiles	Comparison of groups	High NLR was associated with poor overall survival, but not with disease free survival.
2016	Zeng et al. ^31^	115 locoregionally advanced LSCC	Prognostic evaluation	Progression free survival, overall survival	Median ratio	3	NLR was a prognostic factor for overall- and progression free survival.
2017	Kara et al. ^32^	81 LSCC	Prognostic evaluation	Progression free survival, overall survival	Receiver Operating Characteristic (ROC) Curve Analysis	2.04	High NLR values indicated a high risk in local recurrence and decrease in progression free survival.
2017	Wang et al. ^33^	120 LSCC	Prognostic evaluation	Recurrence free survival, overall survival	Receiver Operating Characteristic (ROC) Curve Analysis	2.79	NLR was a prognostic factor for overall- and recurrence free survival.
2017	Hsueh et al. ^34^	979 LSCC	Prognostic evaluation	Disease free survival, Disease specific survival	Tertiles	<1.62, 1.62–2.40, >2.40	High NLR value (>2.40) was an independent prognostic factor for disease free- and disease specific survival.

Receiver Operating Characteristic (ROC) Curve Analysis.

### Neither NLR nor dNLR was a diagnostic biomarker for laryngeal tumors

This study demonstrated that both NLR and dNLR values were not statistically different between patients with benign, premalignant and malignant neoplasms, even though high values were detected in patients with LC (Fig. 1). In contrast, Kum et al. and Duzlu et al.^27^, ^28^ reported a statistically significant difference between patients with LC and other groups when the NLR values were compared. The study populations might be the major reason for this difference. It is obvious that patients with advanced-stage LC had remarkably high NLR and dNLR values when compared with patients with early-stage LC (Fig. 1). Inconsistent with our data, Wong et al.^30^ reported significantly higher values of NLR in patients with advanced-stage LC. As a matter of fact, a statistically significant difference might be determined when a study population that predominantly enrolled patients with advanced-stage LC.

In this study, standardized cutoff values were also preferred in order to stratify the study population and increase the quality of assessment. A cutoff value of 4 for NLR and 2 for dNLR were particularly selected depending on recent literature.^5^, ^6^ A systematic review and meta-analysis, in which a cutoff value of 4 was selected, reported that patients with NLR ≥ 4 had poor OS in several solid tumors.^5^ Moreover, Proctor et al.^6^ evaluated the prognostic role of NLR and dNLR in several cancers including head and neck cancers; and emphasized that optimal thresholds for NLR and dNLR were 4 and 2, respectively. Of note, we were unable to demonstrate a statistically significant difference between patients with benign, premalignant and malignant neoplasms even after the stratification of the study population according to abovementioned cut-off values (Table 1).

### Neutrophil–lymphocyte ratio and dNLR were predictive biomarkers for TCI, LNM, N stage and M stage

In patients with LC, the assessment of TCI, LNM, and DM is of utmost important for accurate clinical staging and treatment selection. Our study demonstrated that both NLR and dNLR were highly correlated with TCI, LNM, T stage, N stage, and M stage (Table 1) (Fig. 1). Similarly, Liu et al.^35^ determined a statistically significant relationship between NLR value and tumor size in patients with thyroid cancer. In our study, high NLR and dNLR values were also detected in patients with transglottic and supraglottic LC when compared with glottic LC. As a matter of fact, patients with transglottic/supraglottic tumors generally have locoregionally advanced LC and poor survival when compared with patients with glottic tumors.^36^ Therefore, all of the aforementioned results demonstrated that both NLR and dNLR are likely to be associated with local tumor extension, regional and distant disease, and may indicate the severity of larynx cancer.

### NLR, not dNLR, was a prognostic factor for 5 year OS, DFS, and LRRFS

In this study, patients with high NLR values (≥4) had a poor prognosis (Fig. 2A–C). Multivariate analysis also determined that NLR was an independent prognostic factor, and more than two-fold increase in the risk of mortality was determined in patients with high NLR value. In the literature, only a few numbers of clinical studies evaluated the prognostic role of NLR in patients with LC, and all of them used different cut-off values for risk stratification (Table 3). In this study, cut-off values of 4 for NLR and 2 for dNLR were particularly preferred due to the abovementioned scientific reasons. Inconsistent with our data, Tu et al.^29^ determined that NLR was a prognostic factor for both OS and DFS. In addition, Zeng et al. detected a statistically significant relationship between high NLR values and both OS and progression free-survival in patients with locoregionally advanced LSCC treated with chemoradiotherapy.^31^ Interestingly, Wong et al.^30^ reported that high NLR value was only correlated with poor OS, not with DFS. Recently, Kara et al. reported that NLR predicted local recurrence and decreased PFS.^31^ In addition, Wang et al.^33^ and Hsueh et al.^34^ showed the prognostic role of NLR in LC (Table 3).

In our study, no statistically significant difference was determined in oncological outcomes when a comparison according to high and low dNLR values was performed (Fig. 1). In addition, uni- and multivariate analysis demonstrated that a high dNLR value did not lead to an increase in the risk of mortality (Table 2). Similarly, Dirican et al.^37^ determined that NLR, not dNLR, was a prognostic factor for patients with breast cancer. In addition, Proctor et al.^6^ emphasized that NLR was a better biomarker to demonstrate prognosis in patients with cancer when compared with dNLR.

## Conclusions

Neutrophil–lymphocyte ratio predicts the oncological outcomes such as LRC, DFS, and OS in patients with LC. As NLR is easily obtained and measured, cost-effective and stable with age and gender, it can be used routinely as a prognostic biomarker in LC. Further prospective studies with standardized cut-off values are required for the risk stratification and determination of prognosis and survival in LC.

## Conflicts of interest

The authors declare no conflicts of interest.
